# Peripheral Dendritic Cells and CD4+CD25+Foxp3+ Regulatory T Cells in the First Trimester of Normal Pregnancy and in Women with Recurrent Miscarriage

**DOI:** 10.1371/journal.pone.0124747

**Published:** 2015-05-06

**Authors:** Maciej Kwiatek, Tomasz Gęca, Arkadiusz Krzyżanowski, Agnieszka Malec, Anna Kwaśniewska

**Affiliations:** Department of Obstetrics and Pathology of Pregnancy, Medical University of Lublin, Lublin, Poland; Otto-von-Guericke University Magdeburg, Medical Faculty, GERMANY

## Abstract

The development of pregnancy is possible due to initiation of immune response in the body of the mother resulting in immune tolerance. Miscarriage may be caused by the impaired maternal immune response to paternal alloantigens located on the surface of trophoblast and fetal cells. The aim of the study was to compare the population of circulating dendritic cells (DCs) and CD4+CD25+Foxp3+ regulatory T cells (TREGs) in the first trimester of a normal pregnancy and in women with recurrent miscarriage and an attempt to determine the relationship between these cells and the role they may play in human reproductive failures. The study was conducted in a group of 33 first trimester pregnant women with recurrent miscarriage and in a group of 20 healthy pregnant women in the first trimester of normal pregnancy. Among mononuclear cells isolated from peripheral blood, the populations of DCs and TREGs were assessed by flow cytometry. The percentage of myeloid DCs and lymphoid DCs showed no significant difference between study and control group. Older maternal age and obesity significantly reduced the pool of circulating myeloid and lymphoid DCs (R=-0.39, p=0.02). In miscarriages the percentage of circulating TREGs was significantly lower compared to normal pregnancies (p=0.003). Among the analysed factors the percentage of TREGs was the most sensitive and the most specific parameter which correlated with the pregnancy loss. The reduction in the population of circulating TREGs suggests immunoregulatory mechanisms disorder in a pregnancy complicated by miscarriage.

## Introduction

Miscarriage is one of the most common complications of pregnancy. The loss of pregnancy before 22 weeks of its duration affects approximately 15–20% of clinically confirmed pregnancies, but the actual percentage of early pregnancy loss has not been precisely determined [1]. Recurrent miscarriage, defined as the loss of three or more consecutive pregnancies, affects about 1–2% of couples [2]. The causes of recurrent miscarriages include (apart from genetic, anatomical, infectious, hormonal or metabolic factors) disorders of auto- and alloimmune origin. However, in about 40–50% of miscarriages no cause can be identified and such cases are classified as idiopathic [3]. This group may include a variety of disorders of maternal immune response to paternal alloantigens located on the surface of trophoblast and fetal cells. The mechanisms allowing mother to maintain the alloantigenic pregnancy and fetus to develop properly are still the subject of many studies. For many years pregnancy was considered a state of immune suppression. According to modern knowledge, the maintenance of pregnancy is possible due to immunological tolerance [4–5].

CD4+ T cells exhibiting a constant expression of interleukin-2 (CD25) α chain receptor are responsible for maintaining peripheral immunological self-tolerance and are called regulatory T cells (TREGs) [6]. It is a heterogenous group of cells, the best known of which are lymphocytes T CD4+CD25+ expressing the transcription factor Foxp3. Such cells are characterized by high expression of CD25. Forkhead box P3 (FoxP3) expression in TREG cells is believed to be a critical factor in the maintenance of TREG cells suppressive function [7]. These lymphocytes differentiate in the thymus into so-called natural TREGs. However, under the influence of cytokines or after contact with an antigen the regulatory T cells may also be formed peripherally [8,9]. Multiple mechanisms of action of TREGs have been proposed, including cell contact dependent and cytokine dependent mechanisms. Dendritic cells (DC) are the cells responsible for professional antigen presentation to T cells and play a significant role in activation, polarization and regulation of the immune response [10]. Two main subtypes of dendritic cells can be distinguished in human blood: myeloid DCs (mDCs) and lymphoid/ plasmacytoid DCs (pDCs). mDCs are the main subgroup in peripheral blood involved in stimulation of T cells. pDCs are components of the innate immune system, produce large amounts of type I interferon. Upon subsequent activation these cells can promote Th2 type immune response. Circulating dendritic cells do not have all the typical features of their counterparts in tissue and they are less mature. Recruitment, activation and expansion of T cells depend on the maturity of DCs. The differentiation of CD4 + T cells occurring in the presence of immature dendritic cells results in stimulation of the immune response and developing immune tolerance [11]. The naive T cells acquire the ability to express the CD25 molecule and Foxp3 factor under the influence of interleukin-10 (IL-10) and with participation of transforming growth factor-β (TGF-β) [12,13]. The ability to maintain the peripheral immune tolerance makes the dendritic cells crucial for maintaining immune homeostasis during pregnancy.

The aim of the study was to compare the population of circulating dendritic cells and CD4+CD25+Foxp3+ regulatory T cells in the first trimester of a normal pregnancy and in women with recurrent miscarriage and an attempt to determine the relationship between these cells and the role they may play in human reproductive failures.

## Materials and Methods

### Selection of patients

The project received approval from the Bioethics Committee of Medical University of Lublin. Written informed consent have been obtained from the participants. The research was conducted in 2012–2013 on patients of the Obstetrics and Pathology of Pregnancy Department, Medical University of Lublin. The study did not include women with a history of any chronic disease nor women with known anatomical anomalies of the womb. Women who became pregnant after use of any assisted reproductive technologies were not included. Women with genetic or infectious etiology of previous miscarriage were excluded. The study included 70 women in the single pregnancy between 6th and 13th gestational weeks. The study group consisted of patients with a history of two or more miscarriages who did not deliver any child and experienced another first trimester idiopathic miscarriage in subsequent pregnancy. Gestational age was calculated based on the date of the last menstruation and confirmed by transvaginal ultrasound examination at 6th gestational week. Diagnosis of miscarriage was based on vaginal bleeding and abdominal pain which resulted in fetal products expulsion. Miscarriage was confirmed by ultrasound. The study group was evaluated within 24 hours after the beginning of bleeding. The control group consisted of healthy primiparas from Outpatient Clinic without any complications in the first trimester nor in further stages of pregnancy who delivered at term. The control patients were evaluated during their first obstetrical examination. Height and weight were used to calculate body mass index (BMI) of the pregnant females.

### Isolation of mononuclear cells from the peripheral blood

The material for the study was venous blood collected from the basilic vein. Mononuclear cells were isolated from the blood within 1 hour from sampling. Separation was performed by density gradient centrifugation using Gradisol L (Aqua Medica ZPAM-KOLASA GP, Lodz, Poland) with the acceleration of 600 x g for 20 minutes at room temperature. Subsequently, the layer of mononuclear cells was collected from the interphase and washed twice.

### Phenotyping of peripheral dendritic cells

The isolated cells were suspended in phosphate-buffered saline without Ca^2+^ and Mg^2+^ (PBS; Sera and Vaccines Manufacturer, Biomed, Lublin, Poland) supplemented with 20% human serum albumin (Baxter AG, Vienna, Austria). To thus prepared cell suspension, FcR Blocking Reagent was added (Miltenyi-Biotec GmbH, Bergisch Gladbach, Germany). Labeling of surface antigens was performed by adding monoclonal antibodies conjugated with certain fluorochromes: Mouse anti-human-FITC cd1c (Miltenyi-Biotec, Bergisch Gladbach, Germany), FITC-BDCA2 (Miltenyi-Biotec, Bergisch Gladbach, Germany), CD19-CyChrome (Pharmingen, USA) and CD123-PE (Becton Dickinson, USA). The monoclonal antibodies were added according to the manufacturer's instructions and incubated for 10 minutes at 4°C without exposure to light. The cells were then washed by centrifugation at 300 x g in PBS supplemented with 20% human serum albumin and 2mM EDTA (Sigma, USA) for 10 minutes at 4°C.

### Phenotyping of peripheral CD4+CD25+Foxp3+ regulatory T cells

The isolated mononuclear cells, used to determine the presence of regulatory T cells, were suspended in a medium consisting of 20% human serum albumin (Baxter AG, Vienna, Austria) and RPMI 1640 medium with L-glutamine and NaHCO3 (Biomed, Lublin, Poland). Subsequently, cryoprotective agent—10% DMSO (Sigma-Aldrich, USA)—was added. The prepared cell suspension was frozen at -76°C. Thus secured peripheral blood mononuclear cells were stored until cytometric analysis.

Prior to further analysis, cells were thawed and washed twice in PBS and then filtered. Initially, surface antigens CD4 and CD25 of mononuclear cells were assayed by incubating the cells for 20 minutes at room temperature with anti-PE-Cy5-anti-human CD4 antibodies (BioLegend, San Diego, CA, USA) and PE anti-human CD25 antibodies (BioLegend, San Diego, CA, USA). For cell washing Staining Buffer Cell, Foxp3 Fix/Perm and Perm-buffer were used (BioLegend, San Diego, CA, USA). The cells were stained for intracellular Foxp3 using monoclonal antibody Alexa Fluor 488 anti-human FOXP3 (BioLegend, San Diego, CA, USA). The incubation lasted 30 minutes at room temperature without exposure to light. After incubation, cells were washed in Cell Staining Buffer by centrifugation for 5 minutes at 250 x g.

### The analysis of peripheral blood dendritic cells and CD4+CD25+Foxp3+ regulatory T cells populations wit the use of flow cytometry

The evaluation of peripheral blood dendritic cells and CD4+CD25+Foxp3+ regulatory T cells population was conducted using FACSCalibur flow cytometer (Becton Dickinson, USA). The acquisition and analysis of the results was performed using FacsDiva. During the analysis of DCs at least 300 000 cells were gated from each sample. During the analysis of TREGs at least 100 cells were collected from each sample. The intensity of the light scattered by cells and projected in the forward direction just off the axis of the laser beam (FSC, forward scatter) and the intensity of light scattered by the granularities of the cells at 90 degrees to the laser beam axis (SSC, side scatter) were evaluated.

**Fig 1:**
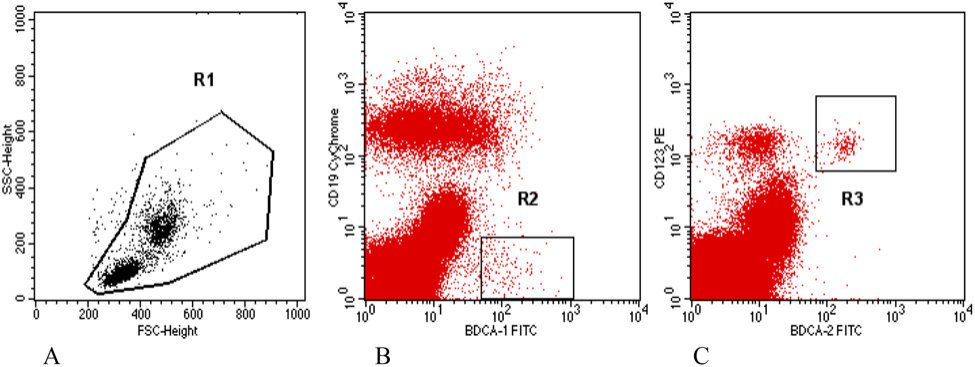
Cytometric analysis of myeloid and plasmocytoid dendritic cells among peripheral mononuclear cells in a woman with miscarriage. The percentage of DCs was assumed among mononuclear cells of the section R1 (Fig 1A). Section R1 was based on FSC and SSC. BDCA-1+CD19- cells from section R2 were rated as circulating mDCs (Fig 1B). BDCA-2+CD123+ cells were rated as circulating pDCs (Fig 1C).

Mononuclear cells were differentiated on the basis of color analysis, and the results were presented as a percentage of positive cells in the test sample. Differentiation between mDCs and small, residual B cells, both of which express the antigen BDCA-1 (CD1c), was performed by analyzing the presence of the CD19 antigen. CD1c+CD19+ B cells were eliminated from further analysis. Only the cells with CD1c+ CD19- phenotype were classified as circulating mDCs. Among the mononuclear cells, the cells expressing BDCA-2 and CD123 antigens were classified as circulating pDCs (Fig 1).

**Fig 2:**
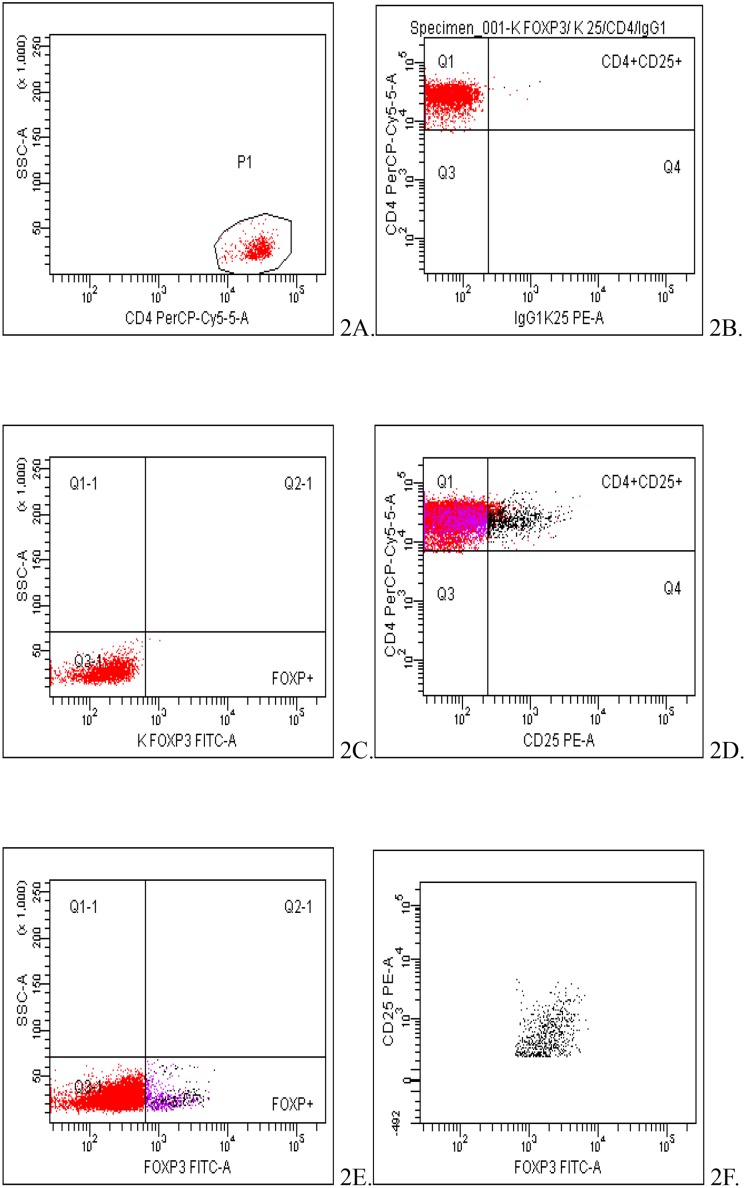
Cytometric analysis of peripheral blood T regulatory cells (CD4+CD25+Foxp3+) of a patient at the 10th week of normal pregnancy. The evaluation of TREGS percentage was conducted among CD4+ cells in P1 population (Fig 2A). Fig 2B-C show negative controls of the sample. Fig 2D-E show the helper cells (CD4+) expressing CD25 antigen (Fig 2D) and Foxp3 antigen (Fig 2E). Fig 2F shows the population of TREGs cells (CD4+ CD25+ Foxp3+) in the test sample.

Regulatory T cells were identified as cells of CD4+CD25+Foxp3+ phenotype (Fig 2).

### Statistical analysis

The values of the analyzed measurable parameters were characterized with the use of mean, median and standard deviation and the values of the immeasurable parameters using amount and percentage. The normality of the distribution of the analyzed parameters for the measurable features was evaluated using W Shapiro-Wilk test.

U. Mann- Whitney test was used to compare two independent groups. Kruskal-Wallis test was used to compare the age of the groups. R. Spearman correlation was used to examine the relationship between the variables. Chi-square test for homogeneity was used for unrelated qualitative features to detect the existence of differences between the compared groups. Chi-square test for independence was used to determine the existence of relationship between the measured features. Logistic analysis and ROC curves were used to assess the risk of miscarriage.

P-values <0.05 were considered as statistically significant. Statistical analysis was performed using STATISTICA 10.0 software (StatSoft, Poland).

## Results

From the group of 70 women involved in the study, 17 were excluded from further analysis, including 10 pregnant women from the study group (2 cases of uterine septum, 2 cases of balanced translocation in a partner, 3 cases of antiphospholipid syndrome, 3 bleedings not followed by miscarriage) and 7 from the control group (5 cases of spontaneous miscarriage, 2 cases of early preeclampsia). Information concerning maternal age, gestational age and obstetrical history has been presented in Table 1.

**Table 1:** Characteristics of study and control cohort.

	Control group	Study group	All	*p* value
**Maternal age *(years)***	27.30±2,79	30.70±4,54	29.42±4,28	
**Gestational age *(gestational weeks)***	10.2	9.6	10.09	0.47
**Nulliparas**	n = 20 (37.74%)	n = 33 (62.26%)	n = 53 (100%)	
**Number of miscarriages in anamnesis**	n = 0	*two* n = 20 (60.61%)		
		*three* n = 9 (27.27%)		
		*four* n = 4 (12.12%)		

### Dendritic cells

**Fig 3:**
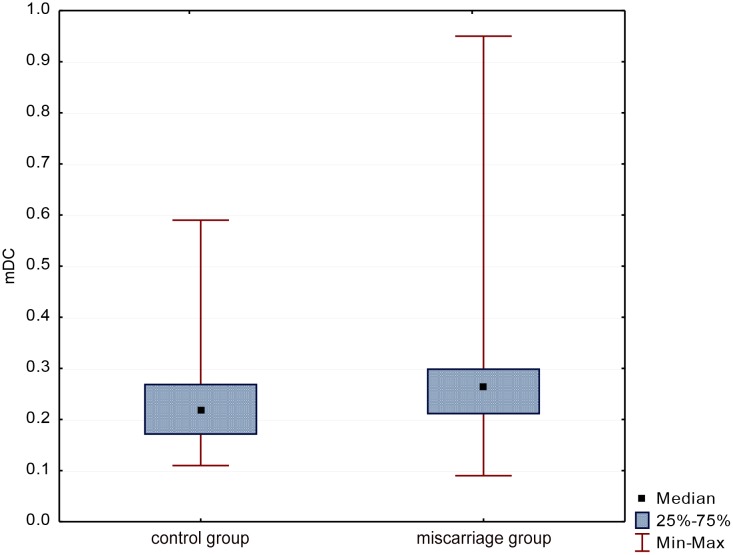
Evaluation of mDCs percentage in groups.

The research found that the percentage of mDCs in the whole group of women was 0.25 ± 0.13 (Me = 0.23). The analysis showed that in women with miscarriage the percentage of mDCs was slightly lower compared to the control group (0.23 ± 0.09 vs. 0.28 ± 0.18 respectively). These differences were not statistically significant (p = 0.17), (Fig 3).

**Fig 4:**
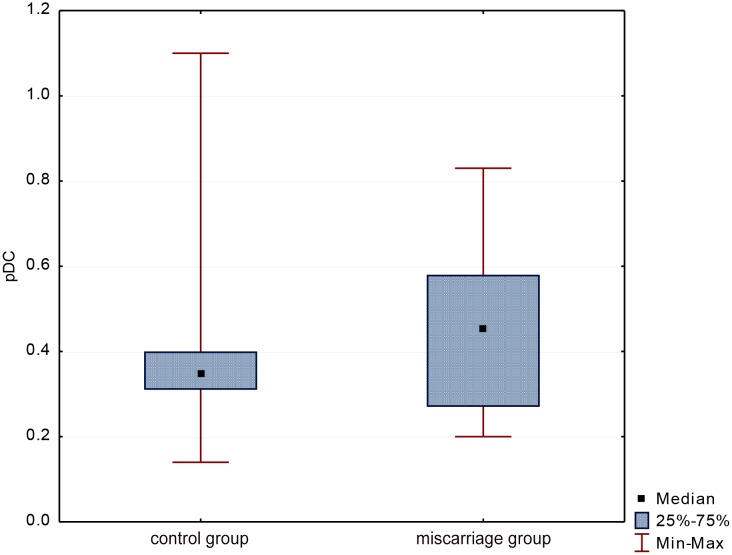
Evaluation of pDCs percentage in groups.

The average percentage of pDCs in all women was 0.42 ± 0.19 (Me = 0.36). Statistical analysis showed that the percentage of these cells was slightly lower (p = 0.34) in women with miscarriage than in the control group (0.40 ± 0.19 vs. 0.45 ± 0.19 respectively), (Fig 4).

### TREG cells

**Fig 5:**
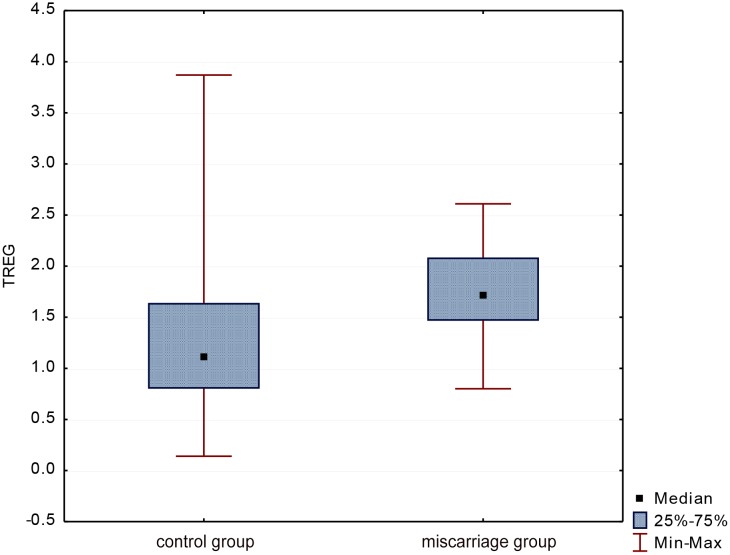
Evaluation of TREGs percentage in groups.

The average percentage of TREGs in the whole group was 1.51 ± 0.71 (Me = 1.49). Statistical analysis showed that the percentage of TREGs was significantly lower (p = 0.003) in women with miscarriage than in the control group (1.33 ± 0.76 vs. 1.80 ± 0.51 respectively), (Fig 5).

### Relationship between percentage of cells and maternal age, gestational age, BMI

**Fig 6:**
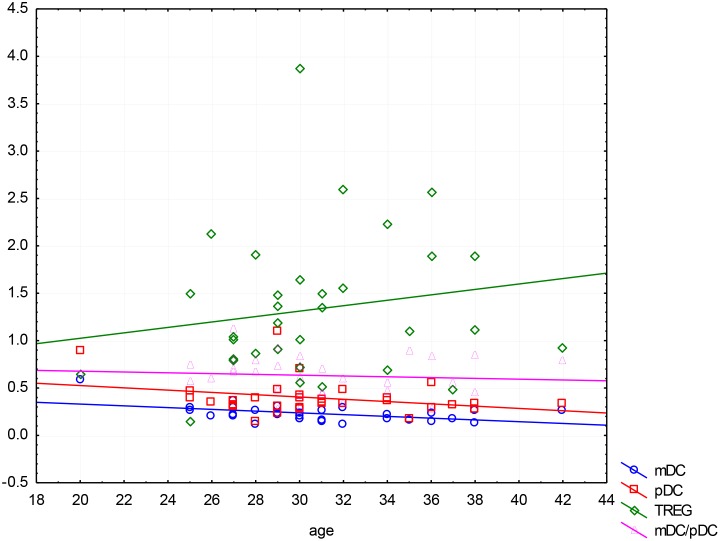
Correlation between maternal age and the percentage of mDCs, pDCs and TREGs in patients with miscarriage.

In women with miscarriage the correlation analysis found a significant association between age and the percentage of mDCs, (R = -0.39, p = 0.02). With age, the percentage of cells decreases. There was no relationship between age and other cells both in the group of miscarriage, as well as in the control group (p > 0.05), (Fig 6).

**Fig 7:**
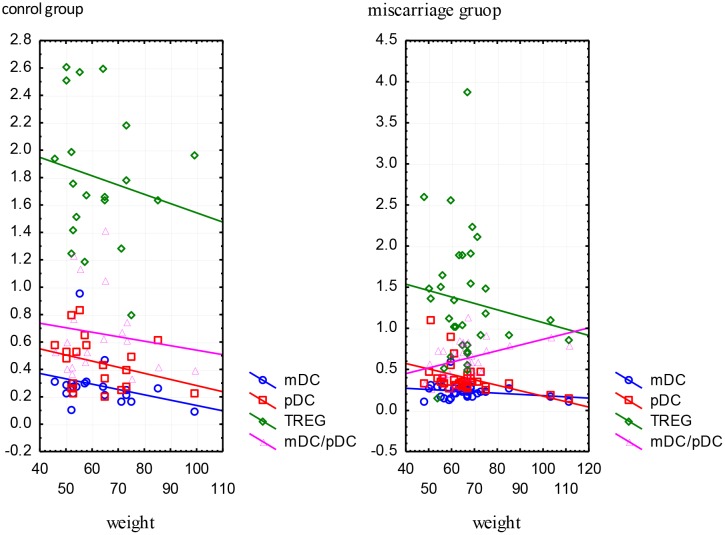
Correlation between body weight (kilograms) and the percentage of mDCs, pDCs, TREGs and mDC/pDC ratio in the study and control group.

The study showed no significant relationship between the percentage of cells and gestational age both in women with miscarriage, as well as in the control group (p> 0.05). 62.26% (n = 33) of women had normal BMI, but 13.21% (n = 7) were underweight and 24.53% (n = 13) were overweight. Women with miscarriage were overweight slightly more often in comparison to the control group (27.27% vs. 20.20%) but the difference was not statistically significant, (p = 0,49). In women with miscarriage correlation analysis found a significant association between body weight with a percentage of pDCs (R = -0.39, p = 0.02) and mDC/pDC ratio (R = 0.43, p = 0.01). Increased body weight affects the decrease in the percentage of pDCs and the increase in the mDC/pDC ratio. In women with miscarriage there was no significant relationship between body weight and TREGs and mDCs. In the control group, the relationship of body weight with the percentage of the selected cells was not statistically insignificant (p> 0.05), (Fig 7).

### Miscarriage risk assessment

**Fig 8:**
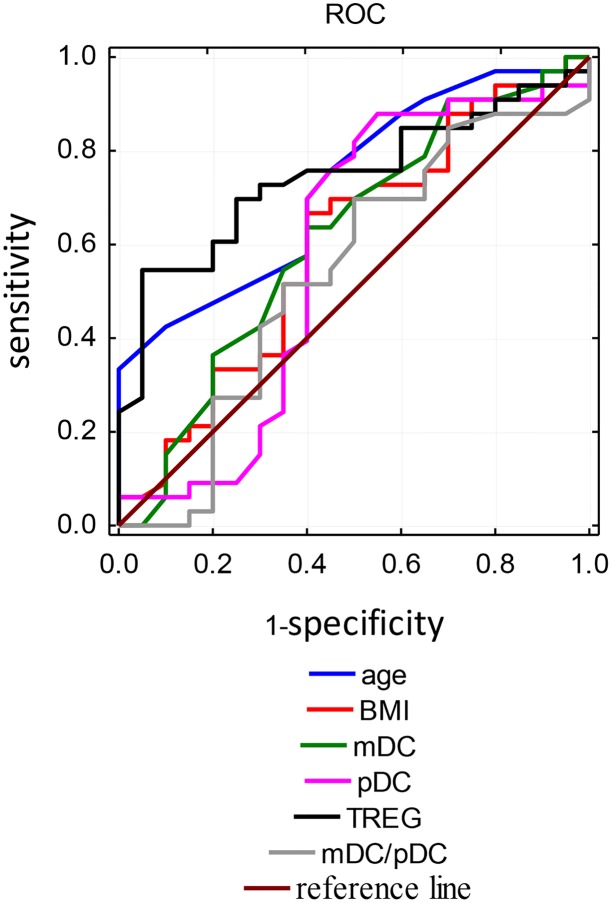
ROC curves for risk factors of miscarriage incidence.

ROC curves were used for evaluation of the factors affecting the occurrence of miscarriage. The analysis included variables such as age, BMI, percentage of mDCs, pDCs and TREGs and mDC/pDC ratio. The percentage of TREGs was the best predictive factor, with a cut-off point of 1.50, and the sensitivity and specificity of this test were respectively 70% and 75. The area under the curve was 0.74 for the TREGs. Other variables such as age, BMI have a high area under the curve, but the specificity was low, at high sensitivity. The results obtained for the other variables are presented in Table 2 and Fig 8.

**Table 2:** The evaluation of the predictors affecting the incidence of miscarriage.

Predictors	AUC	Cut off point	Confidence level - 95%	Confidence level + 95%	Sensitivity	Specificity
Maternal age	0.73	27	0.59	0.86	0.88	0.40
BMI	0.80	19.71	0.44	0.76	0.88	0.30
mDCs	0.62	0.29	0.45	0.78	0.91	0.30
pDCs	0.58	0.48	0.40	0.76	0.88	0.45
TREGs	0.74	1.50	0.61	0.88	0.70	0.75
mDC/pDC	0.54	0.44	0.37	0.71	0.85	0.30

## Discussion

The proper development of pregnancy is possible only with adequate adaptation of the maternal immune system. In 1953, Peter Medawar first time pointed out the relationship between the maternal immune system and fetal antigens [14]. The progress of knowledge allowed disproving some of the theories regarding the placental barrier, insufficient maturity of fetal antigens or a weakening of the immune response during pregnancy. Nowadays it is known that during pregnancy the direct contact occurs between the fetal and maternal cells, and fully mature antigens of the major histocompatibility complex present on placental or fetal cells penetrate into the maternal circulation, where they have the potential ability to induce a strong immune response leading to rejection of the foreign allogeneic tissue [15,16]. Furthermore, the immune system in pregnancy is not in a state of inertia, as recognition of fetal antigens by maternal immune competent cells leads to the development of the cascade of immunological events [17].

Miscarriage, preeclampsia, and intrauterine growth restriction are examples of the pathology of pregnancy, in which the immune factor has a documented participation [18–20]. Dendritic cells, the regulatory T lymphocytes as well as other elements of the immune system are certainly important in promoting maternal tolerance towards the fetus. These issues are an important topic of interest to many researchers.

Incomplete activation of peripheral DCs seems to be the most important feature of these cells in normal gestation. The markers of the incomplete activation enhance with the development of pregnancy. It involves increased expression of co-stimulatory molecules and maturation markers, whereas no increase in the expression of human leukocyte antigen-DR (HLA-DR). Precisely the lack of overexpression of HLA-DR can be crucial to the maintenance of tolerance towards the fetus. In the case of noxious agent such as pathogens or tissue damage, HLA-DR expression on the surface of DCs is always accompanied by the expression of co-stimulatory molecules and cytokine production which aims to increase the number of the MHC—protein complexes presented to T cells [21]. Intense activity of dendritic cells in both peripheral blood and decidua may thus lead to an inflammatory response that may adversely affect pregnancy.

In pregnant increased mDCs/pDCs ratio in the peripheral blood is reported. This is due to the increase in the population of mDCs observed both in blood and in the decidua. Recruitment of this fraction of cells and their peripheral location are likely due to the interaction between hormones, growth factors and cytokines [22–23].

In this paper a statistical analysis of peripheral dendritic cells populations showed that in cases of miscarriage the percentage of mDCs and pDCs was slightly lower than in normal pregnancies, but these differences did not show statistical significance. It is suggested that the reduction in the population of dendritic cells in peripheral blood in miscarriage results from their migration into peripheral tissues, wherein influencing the synthesis of pro-inflammatory cytokines are involved in the fetal rejection. Interesting results have also been obtained by correlating the percentage of DCs with maternal age, and body weight. Obtained statistically significant differences suggest that older maternal age, and obesity significantly reduce the pool of circulating mDCs and pDCs. Perhaps the maternal age decreases the tolerogenic response of the immune system, and obesity can result in weakening of Th2-type immune responses and ultimately lead to miscarriage.

Age-dependent reduction in a subpopulation of mDC (CD11b+ Gr-1/Ly6C+) in mice has been reported [23]. The researchers found that impaired T cells differentiation and their dysfunction may be a result of changes in the profile of DC in older adults. The consequences of aging on TLR function in human mDCs and pDCs remain incompletely understood. It was stated that alterations in TLR expression and signaling in DCs, combined with other age-related findings in monocytes and macrophages from aging individuals, such as decreased co-stimulatory protein expression, could further diminish B and T cell function [24,25].

Circulating DCs are significantly decreased in obese non-pregnant adults. However, obese DCs produce twice more of the immunosuppressive cytokine interleukin IL-10 than lean controls, and in turn stimulate four times more IL-4-production from allogeneic naive T cells [26]. Obesity may negatively impact the ability of DCs to mature and elicit appropriate T-cell responses to a general stimulus.

Studies in the available literature indicate that most researchers focus on decidual dendritic cells and their role in recurrent miscarriage. There are almost no reports of DCs circulating in peripheral blood in women with spontaneous nor recurrent miscarriage.

Studies on the role of dendritic cells in miscarriages conducted by Tirado-González et al. aimed at comparing the decidual DC-SIGN+ cells in pregnancies complicated by miscarriage and normal pregnancies undergoing elective termination between 6th and 11th gestational week [27]. It was found that in normal pregnancies decidua was dominated by dendritic cells of myeloid immature DC phenotype. In aborted tissues the majority of DC-SIGN+ cells were of CD14+ phenotype corresponding to macrophages, and the percentage of dendritic cells was significantly lower. These studies, however, did not fully explain the changes in populations of DC-SIGN+ cells in cases of abortion. The question if a lower percentage of mDCs in miscarriages can be caused by migration of these cells to local lymph nodes, where as mature mDCs induce a Th1 response, could be answered by the study of the lymph nodes, which, however, is limited in women for ethical reasons.

Dendritic cells at the maternal-fetal interface contribute to the promotion of maternal tolerance by various mechanisms. The most important appears to be the induction of T regulatory cells capable of suppressing maternal effector T cells. Environment created by cytokines appears to play a key role in determining the phenotype of dendritic cells and in regulation of intensity signals sent to regulatory T cells. DCs, which differentiate in the presence of TGF- ß, IL-10, GM-CSF and IL-4, have immunotypes and functional characteristics of immature cells that induce CD4+CD25+Foxp3+ T cells. Similarly microenvironment in which the naive CD4+ T cells contact with an antigen, has a great impact on the differentiation and development of regulatory cells [28].

Since the end of the twentieth century it has been known that pregnant proper development depends on the presence and activity of the CD25+ T cells [29]. It was found that in pregnancies complicated by spontaneous miscarriages a subpopulation of CD4+CD25^high^ T cells is significantly smaller both in the decidua and in the peripheral blood [30,31]. Furthermore, the expression of CTLA-4 on the surface of CD4+CD25^high^ cells, a molecule responsible for inhibition of T cell proliferation and transmission of the inhibitory signals, was increased in normal early pregnancy, and reduced in miscarriage [31]. This proves that the decreased expansion of TREGs during pregnancy, as well as their reduced immunosuppressive properties may predispose to pregnancy loss. In our findings the percentage of circulating CD4+CD25+Foxp3+ T cells was significantly lower in miscarriage in comparison to normal pregnancies (p = 0.003). The results seem to be consistent with other studies and confirm the role of these lymphocytes in reproductive failures [30–32].

The retrospective analysis of various immune factors which may be used as markers of a miscarriage conducted by Winger et al. demonstrated that only the percentage of circulating T regulatory cells achieved a statistically significant difference (p = 0.005) between normal and complicated by miscarriage pregnancies [33]. The authors found that women in the first trimester with TREGs percentage below 0.7% were at significantly higher risk of pregnancy loss, than women with TREGs percentage above 0.7% (44% vs. 80% respectively). At the same time an increase in the percentage of TREGs in subsequent assays was observed two times less frequently in miscarriage compared to normal pregnancies. In our analysis the percentage of DCs and TREGs was evaluated only once, when miscarriage occured. Nevertheless, among the analysed factors the percentage of TREGs was the most sensitive and the most specific parameter which correlated with the pregnancy loss.

The modern research stress a significant role of dendritic cells and regulatory T cells in the supervision of the immune response and maintenance of immune tolerance. Shift of the immune response between suppression and activation appears to be essential in the pathogenesis of numerous diseases, including reproductive disorders. Further studies and knowledge of the physiology and function of dendritic cells and CD4+CD25+Foxp3+ regulatory T cells will allow the development of new diagnostic and therapeutic options based on interference in the number, function, or specificity of these cells.

## Conclusions

The percentage of peripheral myeloid and lymphoid dendritic cells do not show significant difference between healthy first trimester women and women with recurrent miscarriage. Maternal obesity and maternal age may interfere with the immune response towards fetus.

The reduction of the population of circulating CD4+CD25+Foxp3+ regulatory T cells exhibit impaired immune regulatory mechanisms in pregnancy complicated by miscarriage.

The analysis of CD4+CD25+Foxp3+ regulatory T cells population may become a risk assessment test for an abnormal development of pregnancy and miscarriage.
